# Novel Bioluminescent Quantitative Detection of Nucleic Acid Amplification in Real-Time

**DOI:** 10.1371/journal.pone.0014155

**Published:** 2010-11-30

**Authors:** Olga A. Gandelman, Vicki L. Church, Cathy A. Moore, Guy Kiddle, Christopher A. Carne, Surendra Parmar, Hamid Jalal, Laurence C. Tisi, James A. H. Murray

**Affiliations:** 1 Lumora Ltd., Ely, United Kingdom; 2 Institute of Biotechnology, University of Cambridge, Cambridge, United Kingdom; 3 Clinic 1A, Addenbrooke's Hospital, Cambridge, United Kingdom; 4 Clinical Microbiology and Public Health Laboratory, Addenbrooke's Hospital, Cambridge, United Kingdom; 5 Cardiff School of Biosciences, Cardiff University, Cardiff, United Kingdom; Instituto Butantan, Brazil

## Abstract

**Background:**

The real-time monitoring of polynucleotide amplification is at the core of most molecular assays. This conventionally relies on fluorescent detection of the amplicon produced, requiring complex and costly hardware, often restricting it to specialised laboratories.

**Principal Findings:**

Here we report the first real-time, closed-tube luminescent reporter system for nucleic acid amplification technologies (NAATs) enabling the progress of amplification to be continuously monitored using simple light measuring equipment. The Bioluminescent Assay in Real-Time (BART) continuously reports through bioluminescent output the exponential increase of inorganic pyrophosphate (PP_i_) produced during the isothermal amplification of a specific nucleic acid target. BART relies on the coupled conversion of inorganic pyrophosphate (PP_i_) produced stoichiometrically during nucleic acid synthesis to ATP by the enzyme ATP sulfurylase, and can therefore be coupled to a wide range of isothermal NAATs. During nucleic acid amplification, enzymatic conversion of PP_i_ released during DNA synthesis into ATP is continuously monitored through the bioluminescence generated by thermostable firefly luciferase. The assay shows a unique kinetic signature for nucleic acid amplifications with a readily identifiable light output peak, whose timing is proportional to the concentration of original target nucleic acid. This allows qualitative and quantitative analysis of specific targets, and readily differentiates between negative and positive samples. Since quantitation in BART is based on determination of time-to-peak rather than absolute intensity of light emission, complex or highly sensitive light detectors are not required.

**Conclusions:**

The combined chemistries of the BART reporter and amplification require only a constant temperature maintained by a heating block and are shown to be robust in the analysis of clinical samples. Since monitoring the BART reaction requires only a simple light detector, the iNAAT-BART combination is ideal for molecular diagnostic assays in both laboratory and low resource settings.

## Introduction

In recent years the molecular amplification of polynucleotides has become increasingly important in life sciences. Many variants of these technologies exist, and they increasingly underpin commercial diagnostic tests as well as a large number of research applications. Most diagnostic applications rely on detection of a target nucleic acid through the process of amplification whose specificity is determined by the use of oligonucleotide primers complementary to the target sequence. The full potential of these analytical tools is only realised if the analysis can detect, report and quantify the amplification occurring in a closed-tube format in real-time [1]–[3]. Such assays can determine both the presence and concentration of the target in the original sample in a closed-tube format that minimises the risk of contaminating other samples with amplified DNA.

The most common real-time detection solutions utilize fluorescence technologies to report the *in-vitro* synthesis of polynucleotides during the polymerase chain reaction (PCR) [4]. Intercalating dyes and fluorescently-labelled oligonucleotides are the most widely used methods of detection of the ongoing synthesis of the target amplicon, despite their requirement for relatively sophisticated optical equipment to excite the fluorophore of choice and detect the emitted light [5], [6]. Unfortunately, the elaborate nature of such machinery has constrained attempts to produce robust, low-cost instruments.

**Figure 1:**
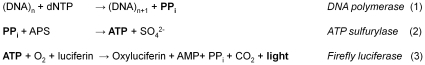
Biochemistry of ELIDA and BART. Schematic biochemical reactions describing nucleic acid amplification (1) coupled with bioluminescent detection of inorganic pyrophosphate using ELIDA (2) and (3).

Alternative approaches of amplification detection have been adopted that determine the production of inorganic pyrophosphate (PP_i_), a low-molecular weight by-product of all polynucleotide amplification [7]–[9]. One molecule of PP_i_ is synthesised each time a nucleotide base is added during the polymerization reaction (Figure 1, equation 1). In any given polynucleotide amplification process, the amount of PP_i_ liberated is therefore proportional to the amount of polynucleotide synthesized and hence the starting template concentration; detected PP_i_ can thus be used to quantify the amount of the original target molecule present in a sample. To date turbidimetry is the only method available for detecting PP_i_ continuously in an ongoing amplification reaction. This method utilises the relative insolubility of the Mg^2+^ salt of PP_i_, which precipitates at high concentrations and can be quantified by monitoring the increasing turbidity of the solution. However, relatively high concentrations of PP_i_ are required and so this approach is limited to isothermal nucleic acid amplification technologies (iNAATs) such as loop-mediated amplification (LAMP) [10], [11] that tend to produce large amounts of PP_i_. There are several distinct iNAATs available as alternatives to PCR, which use strand-displacing polymerases instead of heat denaturation to generate single stranded template, and so have the additional advantage that they run at a constant temperature with concomitantly reduced equipment and energy requirements [12].

Alternatively, PP_i_ can be converted into ATP and quantitatively detected using firefly luciferase in an assay known as ELIDA (Enzymatic Luminometric Detection of Inorganic pyrophosphate Assay) [13] and in Pyrosequencing® [7], [9], [14], [15]. In ELIDA, PPi is converted into ATP by the enzyme ATP sulfurylase utilising the substrate adenosine- 5′-O-phosphosulfate (APS), and the ATP generated is simultaneously utilised by firefly luciferase to oxidise its substrate luciferin with the emission of light (*hv*) (Figure 1, equations 2 and 3) [13]. In Pyrosequencing®, ELIDA is used to detect the instantaneous production of PPi as each single nucleotide base is added step-wise to a polynucleotide chain as it is synthesized base-by-base on a template molecule. For each position on the chain, addition of each of the four bases is attempted until successful addition is determined by PP_i_ production, measured by light output through ELIDA.

DNA amplification reactions have not, however, been successfully monitored in real-time using continuous ELIDA because of a number of obstacles: i) the relatively high temperatures required by most iNAATs (>37°C) are incompatible with the poor thermal stability of firefly luciferase; ii) the inevitable presence of abundant quantities of dATP required for DNA synthesis during amplification leads to high bioluminescent backgrounds, since dATP is an alternative substrate for firefly luciferase; iii) the possible release of additional PP_i_ through non-specific priming and non-specific amplification; and, iv) potential additional contamination with PP_i_ and ATP from the sample.

The availability of recombinant thermostable firefly luciferases tolerant to the typical operating temperatures of most iNAATs (≤65°C) suggested the possibility of direct coupling of bioluminescent detection through a continuous ELIDA reaction to an iNAAT, potentially allowing the continuous determination of amplification in real-time in a single-tube system [16]–[18]. Here it is shown that the use of a thermostable luciferase in a continuously monitored single-tube system with optimised concentrations of ATP-producing enzymes allows the quantitative determination of PP_i_ and hence of the progress of DNA amplification despite the above-mentioned limitations. Such BART (Bioluminescent Assay in Real Time) assays are characterised by a unique kinetic signature, common to several coupled iNAATs tested, that allows not only the real-time detection, but also the quantitation of the nucleic acid target, as well as facile determination of negative samples. The BART signal can be detected using simple instruments capable of controlling a heating block and of detecting the significant levels of light produced using photodiodes or a charge-coupled device (CCD) camera. We confirm the robustness of the coupled iNAAT- BART assays to potentially inhibitory components of clinical samples by presenting the results of a pilot trial evaluating the use of LAMP-BART in *Chlamydia trachomatis (CT)* diagnosis from human urine samples.

## Results

### BART kinetic curves

Among currently available iNAATs, LAMP [19] typically generates high amplicon yields in reactions normally run at around 65°C and has been shown to produce sufficient PP_i_ to be detected either by precipitation as its Mg^2+^ salt or through colorimetry using hydroxy naphthol blue [20]. LAMP has also been shown to produce quantitative results in a real-time fluorogenic assay [21], and was therefore selected for initial investigation of the potential of a coupled bioluminometric assay. LAMP primers were designed as described in Materials and Methods complementary to sequences present on the plasmid of *Chlamydia* and assayed using a plasmid template synthesized to contain this sequence, referred to as *Chlamydia* Artificial plasmid Template (ChAT). Reactions were conducted in a closed one-tube format that contained all enzymes and reagents necessary for both DNA amplification and ELIDA and incubated at 55°C, a temperature selected as suitable for primer annealing, DNA synthesis, conversion of PP_i_ to ATP and light emission, as well as ATP sulfurylase and luciferase stability. Such assays are referred to as LAMP-BART assays.

To carry out LAMP-BART reactions, hardware was assembled as described in Materials and Methods, comprising a programmable heating block simply housed within a commercially available chemiluminescence system (essentially a dark box containing a CCD camera viewing the top of the heating block). Light measurements from the camera were recorded every minute for the field of view and analysed by the attached computer.

**Figure 2:**
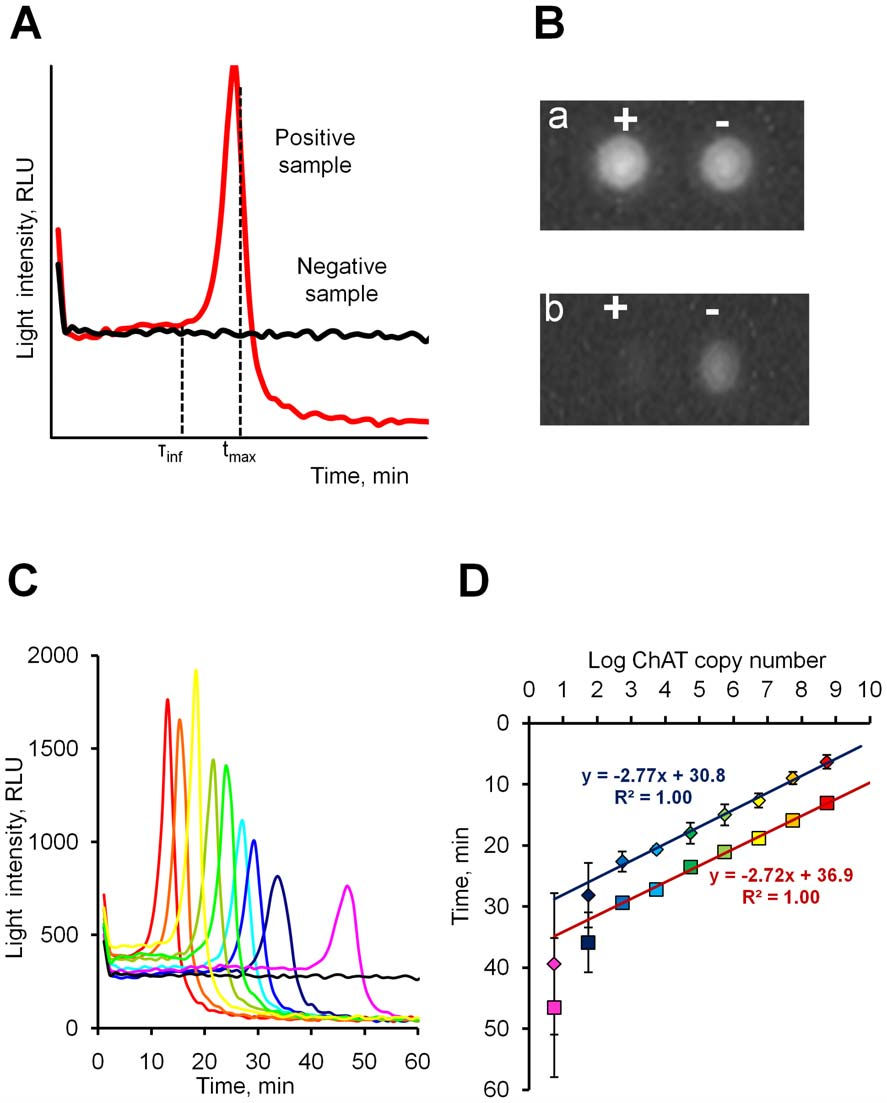
Qualitative and quantitative BART. (A) Typical BART curve (raw experimental data) for a positive sample (red) shows characteristic time to first inflexion point (τ_inf_) and time to peak (t_max_); the curve for a negative sample (black) gradually decays. (B) Images of a positive (+) and a negative (−) samples at the beginning (a) and at the end (b) of the BART assay. (C) Real-time bioluminescent assay of ChAT DNA dilution series amplified by LAMP at 55°C for 1 hour (raw experimental data). 5.5×10^8^ copies – red, 5.5×10^7^ – orange, 5.5×10^6^ – yellow, 5.5×10^5^ – light-green, 5.5×10^4^ – dark-green, 5.5×10^3^ – light-blue, 5.5×10^2^ – dark-blue, 55 – violet, 5.5 – pink, NTC - black. Each curve represents one of three replicates for 5.5×10^3^–5.5×10^8^ copies and one of six replicates for 5.5–5.5×10^2^ measured with or without salmon sperm DNA (100 ng total). (D) Semi-logarithmic plot of the time to first inflexion point (τ_inf_ - blue line) and time to peak (t_max_ – brown line) versus ChAT DNA copy number in the LAMP-BART reactions of which representative curves are shown in (C). Bars show standard deviation. Note that all samples are averaged here, both containing 100 ng carrier DNA or without it.

A profile of light emitted during a positive and a negative ChAT LAMP-BART reactions was recorded over 60 min (Figure 2). A light signal from a negative sample that did not contain any specific template started with a high background and then showed a continual near-exponential decay throughout the reaction. A positive sample had a distinct light output profile characterised by the initially high background decaying for some time in parallel with the negative sample. Unlike the negative sample, however, this initial decay was followed by a rapid increase in light intensity followed by an abrupt decline that diminished below initial baseline levels to an almost undetectable level. This was visualised in a graph of light output against time as a sharp peak of light output (Figure 2A). By the end of the assay, the negative sample maintained a higher light output compared to the positive sample (Figure 2B). Positive ChAT LAMP-BART profiles therefore resulted in highly unusual kinetic curves, very different from the curves reported when LAMP is monitored using either fluorescence or turbidimetry, both of which usually result in sigmoid curves for positive samples, resembling those associated with real-time quantitative PCR (qPCR) [11], [22].

### Effects of ATP, dNTPs, APS and PP_i_ on the light output in BART

There are two major differences between the sigmoid curves described above and the BART curve: the first is the high starting background and the second is the rapid reduction in bioluminescence following the increase that occurs during DNA amplification. To understand further the origin of these differences, we considered the effect on light output in BART of the nucleotides and PPi present in the full reaction.

With respect to nucleotides, the LAMP-BART reaction mixture initially contains high concentrations of all four dNTPs required for nucleic acid amplification as well as the ATP sulfurylase substrate, APS. When a positive sample is amplified in LAMP-BART, it is anticipated that dNTPs will be depleted as PP_i_ is released, APS is converted to ATP through reaction with PP_i_, and ATP is then hydrolysed by luciferase to yield AMP and PP_i_. Therefore, in a positive LAMP-BART assay, a continuous change of concentration of all four dNTPs, APS, PP_i_ and ATP will occur. All these substances with the exception of APS are known to affect firefly luciferase activity [23]–[25] and changes in their levels should therefore have a significant impact on BART light output. ATP and dATP are luciferase substrates, dCTP, dGTP and dTTP are competitive inhibitors of luciferase and PP_i_ may have either a stimulating or inhibitory effect depending on its concentration [24], [25]. To understand better the biochemistry underlying the observed BART curves, so-called ‘deficient’ formulations of ChAT LAMP-BART mixture, containing all ingredients except the primers and Bst DNA polymerase (omitted to prevent any possible specific or non-specific amplification and primer-dimer formation), were investigated with different concentrations of dNTPs, ATP, PP_i_ and APS.

**Figure 3:**
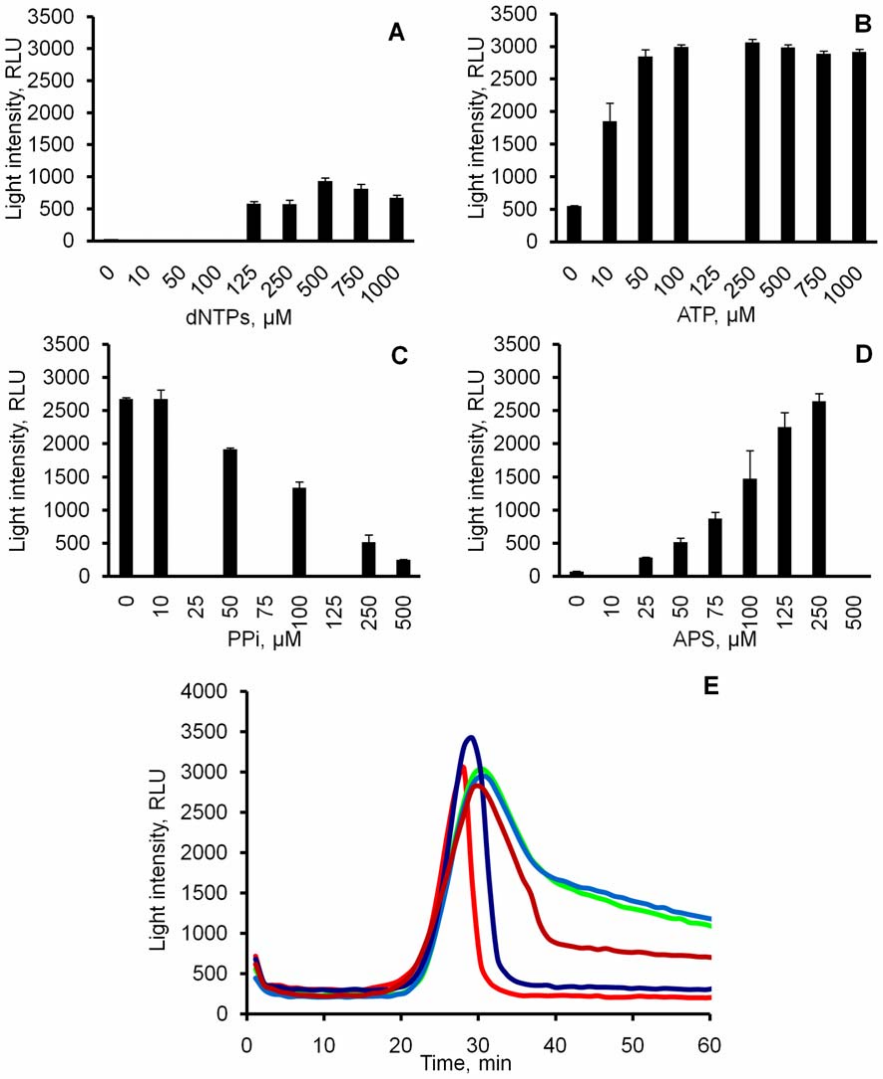
Effect of dNTPs, ATP, PPi and APS on light output in BART. Simulation of effects of different ingredients on the light output in LAMP-BART in a “deficient mix” lacking primers and Bst polymerase but containing all other components as described in each case below. (A) Light output detected using varying concentrations of an equimolar mixture of four dNTPs. Light output peaks at 500 µM total dNTP concentration. (B) Light output detected using varying concentrations of ATP in the presence of 250 µM equimolar dNTPs. Light output is higher than in panel (A) and reaches saturation at 100 µM ATP, showing greater sensitivity to ATP. (C) Inhibitory effect of different concentrations of PPi on the light emission in the presence of 250 µM dNTPs and 100 µM ATP. (D) Stimulatory effect of increasing concentrations of APS on the light emission in the presence of 250 µM dNTPs and 100 µM PPi. (E) Effect of different concentrations of APS on BART curves in complete LAMP-BART formulation with 10^7^ ChAT target DNA (red – 100 µM, navy – 200 µM, brown – 500 µM, green – 750 µM, blue - 1000 µM). As APS concentration is increased, there is little effect on peaking time but more PP_i_ is converted to ATP resulting in a lower rate of inhibition of luciferase and a slower “switch off” of light output.

The effect of the equimolar mixture of dNTPs (0–1 mM each) on light output revealed no background light in the absence of dNTPs and substantial amount of light in the presence of dNTPs (125 µM and above), with a plateau reached at concentrations higher than 250 µM (Figure 3A). This is most likely to be due to the saturation of luciferase with all four dNTPs. Among the four dNTPs present in the mixture dATP is the most likely luciferase substrate causing light emission [23]. The level of light signal was similar to the initial background observed in ChAT LAMP-BART assays, with a gradual decay of light closely resembling that seen in the negative ChAT LAMP-BART (Figure 2A) (data not shown). This decay is typical for all bioluminescent assays utilising firefly luciferase in the presence of high concentrations of substrates and is due to the loss of luciferase enzymatic activity through inhibition by the reaction products, as well as to the gradual thermal inactivation of the enzyme [26], [27]. We therefore consider that the initial high light output and gradual decay is explained by the interaction of luciferase with the high concentrations of dNTPs present in a LAMP-BART reaction.

The effect of ATP was evaluated using ‘deficient’ ChAT LAMP-BART mixture containing 250 µM each dNTP and varying concentrations of ATP (0–1 mM; Figure 3B). Increasing levels of ATP caused a substantial further increase in the background followed by steady decay, similar to that described above (Figure 3A). Addition of 10 µM ATP raised light brightness nearly 4-fold, and 100 µM - 6-fold. Further increase in ATP concentration was not accompanied by any significant changes in the light output, suggesting that in these conditions luciferase was saturated above 100 µM ATP (Figure 3B). The overall higher light output in this experiment suggests that, even though there was no depletion of dNTPs, ATP outcompeted dATP as a luciferase substrate and because of its much higher light-producing efficiency caused an increase in the total light output [28]. The analogous situation is likely to explain the “flash” of light in a positive LAMP-BART amplification, where the increase in light is also facilitated by the depletion of dNTPs.

To address the rapid decline in light output after the “flash”, the effect of PP_i_ was evaluated using a ‘deficient’ ChAT LAMP-BART mixture comprising 250 µM each dNTP, 100 µM ATP and varying concentrations of PP_i_ (0–0.5 mM; Figure 3C). Inclusion of 10 µM PP_i_ had almost no impact on the resulting light output from dNTPs and ATP, while 50 µM PP_i_ reduced light by 30%, 100 µM PP_i_ by 50%, and 250 µM brought it down to the level of typical background coming from dNTPs in the absence of ATP; 500 µM reduced it even further (Figure 3C). Hence, in the presence of high PP_i_, light output decreased to the level below that observed with dNTPs alone - a result similar to that observed at the end of a positive LAMP-BART reaction.

These data are consistent with the explanation that in a positive LAMP-BART, when amplification occurs, PP_i_ is produced and converted into ATP, consuming APS. As long as there is sufficient APS to convert PP_i_ into ATP, the latter is made and provides the substrate for light production by luciferase. The rapid accumulation of PP_i_ and its conversion to ATP during the exponential phase of the amplification then leads to a peak in light output (flash). As APS is exhausted, and if amplification continues, free PP_i_ accumulates and inhibits luciferase, as shown above [24], [25]. This implies that APS concentration should thus have a significant effect on the shape of BART curves.

The effect of APS on BART light output was investigated using both ‘deficient’ and full ChAT LAMP-BART formulations. The ‘deficient’ formulation contained 250 µM each dNTP, 100 µM PP_i_ and different concentrations of APS (0–250 µM) but neither Bst polymerase nor primers. The increase in APS concentration in the presence of a fixed concentration of PP_i_ caused an increase in light production due to the formation of ATP (Figure 3D). The overall result was similar to that shown in Figure 3A, when varying amounts of ATP were introduced directly into the system. The highest light level achieved was close to that observed from the direct addition of 100 µM ATP (compare Figure 3B). In the absence of APS, 100 µM PP_i_ strongly inhibited the background light produced by 250 µM dNTPs (Figure 3D).

Further investigations were carried out with a full ChAT LAMP-BART formulation containing Bst polymerase, primers, 250 µM dNTPs, 10^7^ copies of ChAT template per reaction and varying amounts of APS (0–1 mM). In the presence of 100 and 200 µM APS, a rapid and sharp switch-off of the BART flash was observed. At higher APS concentrations, the light output peaks became broader and did not decline below background even after 60 minutes (Figure 3E), suggesting continuing conversion of PP_i_ to ATP. In line with the explanation suggested above, the final concentration of PP_i_ released through the amplification utilising 250 µM each dNTP could potentially reach 1 mM (assuming full utilization of all dNTPs). With APS limited to 100–200 µM, only part of the PP_i_ released would therefore be able to be converted to ATP, with the further PP_i_ accumulation inhibiting luciferase activity and further light output [24], [25]. The rapid “switch-off” observed as the characteristic feature of a positive LAMP-BART curve is therefore likely to result from the build-up of PP_i_, which cannot be converted into ATP once APS is exhausted.

We conclude that the high initial background in BART is due to the high content of dATP with a possible slight contribution from contaminating PP_i_ and ATP. The characteristic “flash” from positive assays results from rapid ATP production, and the subsequent switch-off is a consequence of inhibition with PP_i_, dependent on APS concentration and ATP sulfurylase enzyme activity.

### Quantitative BART

We next sought to investigate whether the timing of the flash peak, defined by the signal switch-off unique to BART, provides potential for quantitative real-time iNAATs. In a defined LAMP-BART formulation, one may expect that the time required for the same amount of PP_i_ to be released by virtue of amplification process to cause a luminescent flash and its switch-off would be dependent on the amount of nucleic acid target present in the assay. Therefore, a direct relationship between the time-to-peak and the starting target concentration might be anticipated.

Quantitative assessment of LAMP-BART was carried out using the ChAT target (5.5–5.5×10^8^ target molecules per reaction) in the presence or absence of 100 ng non-specific salmon sperm carrier DNA (Figure 2). A positive correlation was observed between both the time to peak (t_max_) and time to first inflexion point (t_infl_) with the template abundance. The apparent t_max_ values varied between 12 and 50 minutes and t_infl_ between 5 and 40 minutes, the timings correlating well with template abundance (Figure 2C). A logarithmic analysis of t_max_ and t_infl_ plotted against ChAT copy number reveals a linear correlation over seven orders of magnitude, and down to 55 copies per reaction (Figure 2D). Below 55 copies, template DNA amplification was still reported, but a linear relationship with respect to template concentration was not observed in these conditions. The t_max_ and t_infl_ data achieved for a given copy number were shown to be highly reproducible, although increased variability was observed when lower concentrations of template were amplified. Time to first inflexion point and time to peak are thus directly correlated with target DNA copy number; this is similar to the correlation observed in qPCR between cycle time (Ct value) and DNA template load. Both correlations showed identical gradients with t_infl_ having a 6-minute smaller intercept. Time to peak is easy to define from the raw data output, while calculating t_infl_ requires some additional data processing, although t_infl_ can be used for faster detection or quantitation of the target present in a sample. We also note that the presence of 100 ng/assay (5 ng/µl) of background salmon sperm DNA had no effect on the quantitation of the target DNA, demonstrating that there is no interference between measured BART signal and this amount of exogenous non-specific nucleic acid present in the assay; a key consideration in measuring unknown samples. It also indicates that reduced quantitation at low copy number is not due to absolute DNA concentration.

### Correlation of DNA synthesis, PP_i_ release and light output in LAMP-BART

**Figure 4:**
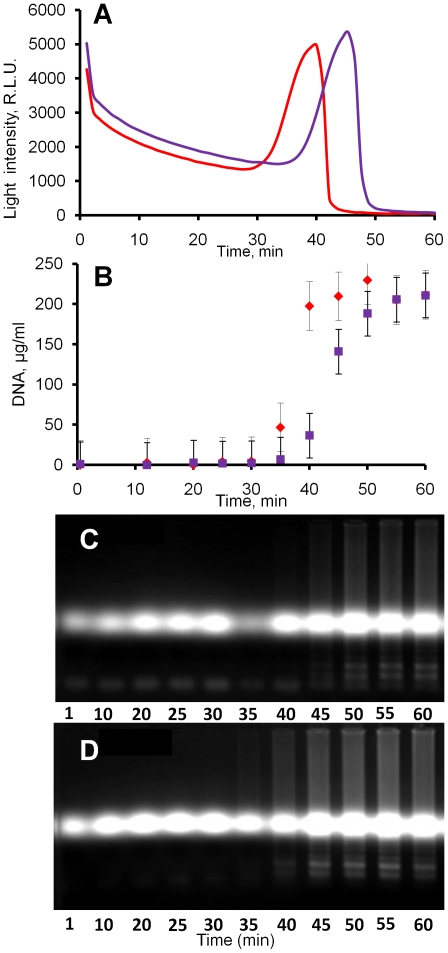
Correlation between bioluminescent output and DNA production in LAMP-BART. Light output in BART (A) and DNA yield assayed by end-point fluorescence method (B) and visualised by gel-analysis (C, D) for two different amounts of ChAT (1 pg/ml – blue, 100 pg/ml - red). Each curve represents one of three replicates. 2% agarose gel shows LAMP amplicon as a ladder of bands representing multiple concatamer repeats of the ChAT template using 1 pg/ml (C) and 100 pg/ml (D) of starting template. The strong band in all lanes corresponds to luciferin, which is strongly fluorescent under UV illumination.

To determine how much DNA and PP_i_ is produced to generate a BART light peak, BART output was monitored in parallel with the independent assessment of DNA synthesis. The real-time bioluminescent output reported during a LAMP-BART reaction with two different starting amounts of the ChAT DNA target (1 pg/ml and 100 pg/ml), was compared to end-point assays for DNA amplification (monitored using fluorescence and gel-analysis), at various time intervals during the assay (Figure 4). Attempted direct end-point measurements of PPi by ELIDA turned out to be unreliable due to the strong interference from the varying concentrations of ATP, APS, dATP and other deoxynucleotides.

In end-point fluorescent DNA analysis, amplicon became detectable at 30 min for the higher target concentration and after 35 min for the lower target concentration and reached 200 µg/ml at approximately 40 and 50 min, respectively (Figure 4B). The corresponding calculated concentration of PP_i_ released by that time in the reaction is approximately 600 µM, a very high level at which APS would already have been depleted and excess PP_i_ would therefore be present in the assay. By gel electrophoresis, LAMP amplicon became visible after 30 min for the higher target concentration and 40 min for the lower, while saturation of the fluorescence from ethidium bromide was reached at 45 and 50 min, respectively (Figure 4C and 4D). Direct comparison of measured amplicon accumulation with the BART signal (Figure 4A) showed that the detectable appearance of amplicon coincided with the time to the first inflexion point in the light output curve. We therefore propose that the increase in light signal started after a prolonged lag-period required for sufficient amplicon to be synthesised and PP_i_ released, at which time nucleic acid amplification became exponential and resulted in the light flash. With the continuing amplification and further release of PP_i_ into the system the light levels of the BART assay then diminished to the lowest point recorded throughout the assay. This corroborates the mechanism proposed above for the strong inhibition of firefly luciferase by free PP_i_, which cannot be converted into ATP because of APS exhaustion [], [24,25]. BART therefore produces a peak of light in real-time when DNA amplification goes into exponential phase.

### Intensity of light output in BART

Unlike conventional bioluminescent assays, BART measurements are intensity-independent. Conventional luciferase bioluminescent assays measure absolute light intensity and correlate its brightness with the levels of the analyte of interest [29]. An attempt to assess and compare the intensity of background light emitted from a ChAT LAMP-BART assay mix using a plate luminometer (BMG) failed, because the photomultiplier was overloaded even when small volumes (down to 5 µl) were measured with the lowest possible voltage setting and shortest integration time (20 ms). Though it was impossible accurately to quantify brightness of BART signals using the plate luminometer, it became clear that BART signals integrated over 60 s could be several orders of magnitude higher than those measured in traditional bioluminescent assays.

**Figure 5:**
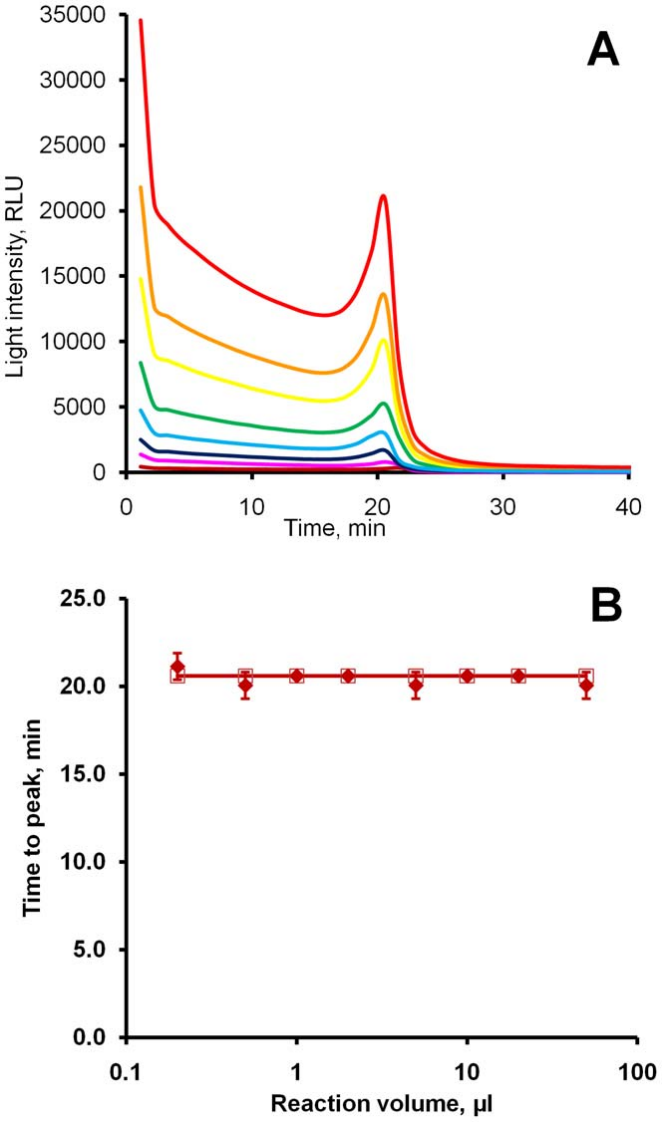
BART output in reactions of different volumes. (A) ChAT LAMP-BART curves recorded at 55°C from reactions of different volumes containing the same concentration of the target. 50 µl – red, 20 µl – orange, 10 µl – yellow, 5 µl – green, 2 µl – light-blue, 1 µl – dark-blue, 0.5 µl – purple, 0.2 µl – brown. Each curve represents one of three replicates. (B) Graph of time to peak against the reaction volume.

BART quantitation is based on temporal parameters, so it is not expected that absolute light output levels affect quantitation. ChAT LAMP-BART amplifications were carried out containing the same concentration of ChAT DNA (10^6^ copies per µl) in different reaction volumes (0.2-50 µl). The intensity of light decreased proportionally with the reduction in volume, but the times to peak remained unchanged, except for a slight increase with the smallest reaction volume (0.2 µl). Reaction volume did not affect either observed t_max_ or peak profile (Figure 5). We therefore conclude that BART quantitation depends on kinetic parameters of the coupled reactions affecting the time to light peak, and not on absolute light output intensity. Taken together with the high level of light signal, this suggests that assays are likely to be tolerant of turbid assay samples, and points to the potential to use low-cost lower sensitivity detection methods for measuring BART light output such as charge-coupled devices or photodiodes.

### Instruments for measuring iNAAT-BART

Unlike the majority of traditional bioluminescent assays, where highly sensitive detection systems are absolutely essential for measuring low-level light, BART produces such bright light outputs that much simpler light detection systems can be employed. Further, since light is emitted from within the reaction mixture itself, no external illumination is required as for fluorescence, and since thermal cycling is not required, substantially simpler hardware can be used to follow BART reactions.

**Figure 6:**
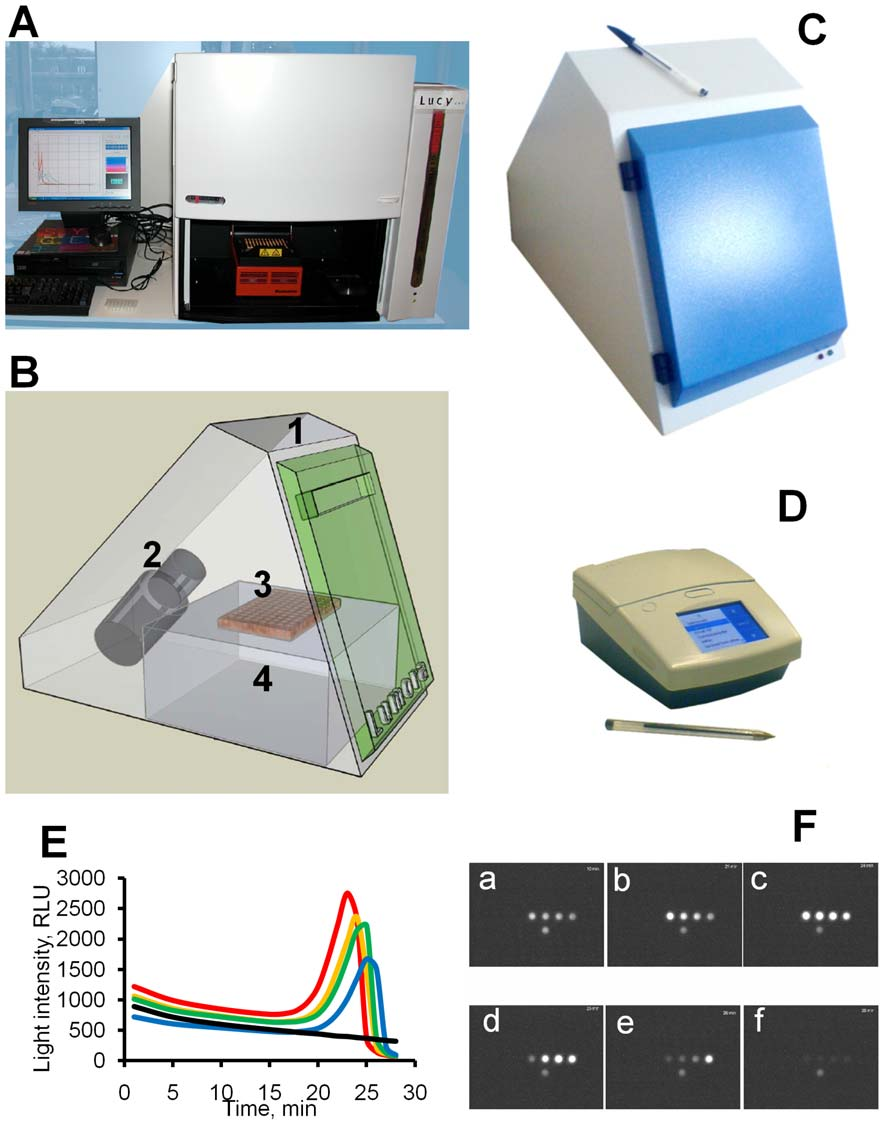
Devices for BART assays and different formats of BART data output. (A) Original laboratory set-up for BART used in the research presented in this paper. (B–D) Later designs of custom equipment for BART assays. High-throughput CCD-camera based system for laboratory use available in 96/384-well format. (B) Exploded diagram and picture (C) of the CCD-camera-based device: 1 – light box, 2 – CCD-camera, 3- samples in standard 0.2 ml PCR tubes or 8-well strips or 96-well plate, 4 – heating block. (D) Portable diode-based device for one or two 8-well strips. (E) Graphical representation of the data for a dilution series: red – 1 ng, orange – 100 pg, green – 10 pg, blue – 1 pg, black - NTC. (F) Corresponding images of LAMP-BART reactions taken at 10 (a), 21 (b), 24 (c), 25 (d), 26 (e) and 30 min (f): top row – ChAT DNA dilution series with the decreasing amount of template 1 ng, 100 pg, 10 pg, 1 pg (left to right); bottom row – no-template control.

Two instruments were therefore designed: a CCD-based detector currently suitable for 96- or 384-well formats, where light reflected by a mirror is detected by a camera from the top of the assay tube (Figure 6B and C), and very small stand-alone photodiode-based 8 or 16-well device, suitable for point-of-use applications or low-resource settings, that reads the emitted light from the bottom of each tube (Figure 6D). Neither machine has moving parts and optical design is simple since the samples do not need to be irradiated. Since quantitation is based upon the measurements of rates of change of light intensity, the need to measure accurately absolute light intensity is much less significant than in conventional bioluminescent assays. BART output can be monitored directly by imaging emitted light (Figure 6F and Movie S1) or represented graphically (Figure 6E). Both instruments utilize algorithms integrated within firmware for data processing to calculate the time to peak and generate a positive-negative call for individual samples by evaluating changes in the rate of light emission. These instruments allow BART to be applied in a wide range of applications from high-throughput screening to point-of-care (POC) and other low-throughput applications.

### Application of LAMP-BART for detection of *Chlamydia trachomatis* in clinical specimens

To assess the application of BART for *in vitro* diagnostics, an evaluation of *Chlamydia trachomatis (CT)* diagnosis in human urine samples was performed, since there is a demand for high sensitivity molecular assays capable of diagnosis at POC [30]. To assess microbial range and selectivity of LAMP-BART for *CT* infection in clinical urine samples, DNA purified from 14 different strains of *CT* was assayed and found to be reliably detected by the ChAT LAMP-BART assay (Table S1). Analytical specificity was assessed using DNA purified from 28 pathogenic bacteria and commensal organisms of the oropharynx and genital tract (Table S2). No false-positives were detected, demonstrating the 100%-specificity of the assay.

Bacterial DNA was isolated as described in Materials and Methods from 105 clinical urine specimens of unknown *CT* status, analysed for *CT* DNA by ChAT LAMP-BART and the results compared to those from qPCR analysis (Table 1). Samples were defined as positive if a light peak was observed within 1 hour in BART and/or had Ct≤40 qPCR cycles. 45 urine samples were diagnosed positive for *CT* by qPCR, of which LAMP-BART reported 43 as *CT*-positive. Importantly, no LAMP-BART false positives occurred. The two samples identified as *CT*-positive only by qPCR had marginal Ct values of 40 cycles. In this comparison, LAMP-BART showed the same specificity as qPCR and 95.6% sensitivity (relative to qPCR). Moreover, it took BART less than 60 min to detect *CT*-positive samples, compared to 120 minutes with the qPCR used. LAMP-BART thus showed robust behaviour with these clinical samples and did not appear to be susceptible to inhibition by potential contaminants present in urine-derived samples subjected to rapid DNA preparation.

**Table 1:** Results of *Chlamydia trachomatis* testing by LAMP-BART and qPCR.

	LAMP-BART	qPCR
Total number of samples	105	105
CT-positive samples	43 (t_max_<60 min)	45 (Ct≤40 cycles)
CT-negative samples	62	60 (Ct>40)
Sensitivity, %	95.6	100
Specificity, %	100	100
Assay time	60 min	2.5 hours
Mean t_max_ or Ct/equivalent time	33.6 min	35.2 cycles ∼1 h 46 min

**Figure 7:**
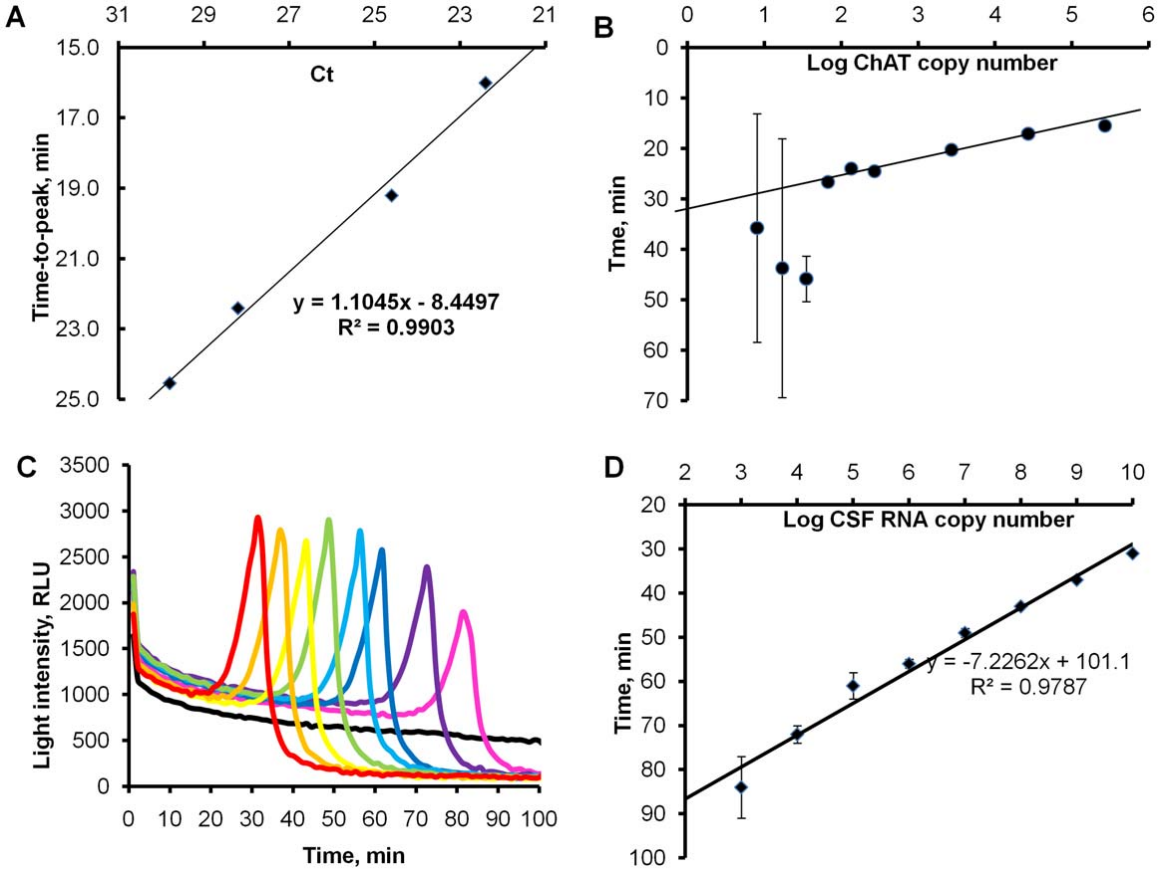
DNA and RNA analysis using LAMP-BART. (A) Correlation between t_max_ values in LAMP-BART (vertical axis) and Ct values in TaqMan PCR (horizontal axis) obtained in the side-by-side analysis of the samples from the *CT* QCMD panel. (B) Effect of increased levels of foreign DNA (1.2 µg salmon sperm DNA/assay) on the sensitivity and speed of ChAT LAMP-BART assay carried out in 26-µl at 60°C. (C) Real-time bioluminescent assay of CSFV RNA fragment dilution series amplified by RT-LAMP at 55°C for 100 min (raw experimental data): 10^10^ copies – red, 10^9^ – orange, 10^8^ – yellow, 10^7^ – green, 10^6^ – light-blue, 10^5^ – dark-blue, 10^4^ – violet, 10^3^– pink, NTC - black. Each curve represents one of three replicates. (D) Semi-logarithmic plot of the time-to-peak versus CSFV RNA copy number in the same RT-LAMP-BART reactions.

A side-by-side comparison of LAMP-BART with a TaqMan PCR currently used for clinical diagnosis [31] by the Health Protection Agency (Cambridge, UK) was carried out using samples from a Quality Control for Molecular Diagnostics (QCMD; http://www.qcmd.org) *CT* panel containing a range of clinically relevant *CT* loads. Accurate *CT* quantification is considered less significant for the clinical management of an infection than reliable detection [31] and in both methods the cryptic plasmid was used as the target for amplification to enhance sensitivity of detection, there being a multiple but variable number of copies of cryptic plasmid in *CT*. Samples were prepared as described in Materials and Methods, and volumes used in LAMP-BART were adjusted to those used in TaqMan PCR to achieve an identical target load in both assays [31]. The samples used and results are presented in Table 2, and correlation between t_max_ values in LAMP-BART and Ct values in TaqMan PCR is shown in Figure 7A. A linear relationship was observed across a wide range of target copy number, two different clinical sampling methods (swabs and urines) and two *CT* variants (Swedish isolate and Dutch isolate) (Table 2). The higher level of potential inhibitors in urine compared to swabs [32] was reflected in the results from urine sample 5, which in spite of a five time higher *CT* load demonstrated later t_max_ and Ct value than the swab sample 4. The linear relationship between t_max_ in LAMP-BART and Ct values in TaqMan PCR supports their similar quantitative ability and the potential use of LAMP-BART for applications requiring quantification of a target. These results also mirror the quantitative nature of real-time LAMP using fluorogenic detection [21].

**Table 2:** Summary of the samples used in the comparison of ChAT LAMP-BART with TaqMan PCR (n/a: not applicable. n/d: not detectable).

Sample	Matrix	CT variant	CT load, cells/ml	Ct	t_max_, min
1	Urine	n/a	0	n/d	n/d
2	Swab	n/a	0	n/d	n/d
3	Swab	Dutch clinical isolate LGV L2	285	29.8	24.5
4	Swab		5700	24.6	19.2
5	Urine		28500	28.2	22.4
6	Urine	Swedish variant	unknown	22.4	16.0

Detection of *CT* cryptic plasmid in clinical samples can be challenged by high levels of additional non-target DNA. Although a total carrier DNA load of 100 ng (equivalent to 5 ng/µl) did not affect LAMP-BART quantification (Figure 2C), the effect of higher levels of DNA on ChAT LAMP-BART was modelled using salmon sperm carrier DNA. A dilution series of ChAT plasmid (4−2.7×10^6^ copies per reaction) was made in 100 ng/µl salmon sperm DNA and analysed under exactly the same conditions as in the side-by-side comparison with TaqMan PCR (Figure 7B). In the presence of 1.2 µg of overall total amount of carrier DNA in the assay ChAT plasmid was detected down to single copies within 50 minutes. Below 70 copies of the target the linearity between the t_max_ and target copy number was lost and reproducibility of the assay was significantly reduced but neither specificity nor sensitivity of LAMP-BART were affected by the presence of large amounts of foreign DNA.

### Application of RT-LAMP-BART for the detection of classical swine fever virus

To demonstrate the applicability of BART to the detection of RNA templates, a model system based on classic swine fever virus (CSFV) was investigated. Purified RNA from an in vitro transcribed artificial template was amplified in a closed-tube one-step format, which included reverse transcription, LAMP amplification and BART detection reagents. For a wide dilution series of RNA (10^3^–10^10^) RT-LAMP-BART resulted in a sequence of light peaks with t_max_ showing inverse linear proportionality to RNA target copy number (Figure 7C and 7D). In the absence of AMV reverse transcriptase, neither amplification nor light peaks were detected, indicating the absence of background DNA and non-specific amplification. BART successfully reported on the exponential release of PP_i_ through amplification of the cDNA copies generated from the RNA target in the coupled assay in the same tube. BART kinetic curves in this coupled RNA-cDNA amplification had exactly the same profile as in DNA amplification and the linear correlation between the starting copy number and t_max_ was retained. This points to the potential for coupled RT-LAMP-BART detection and quantification of RNA viral genome loads for diagnostics in low-resource settings.

## Discussion

Molecular diagnostic tests provide the “gold standard” in terms of sensitivity and specificity, and are in principle capable of detecting single copies of a specific nucleic acid sequence in a sample through the process of repeated copying by nucleic acid amplification. There is a rapidly increasing demand for such molecular diagnostic tests driven by the requirement for sensitive and accurate determination of contaminating or disease organisms, the presence of adventitious genetic material, or the diagnosis of genetically determined disease states. In particular, there is a need for tests providing speed, simplicity and robustness in both molecular assay and the necessary equipment. Such attributes are also vital in the low resource settings of the developing world, where molecular diagnostics have yet to have a widespread impact.

Currently available molecular diagnostic systems are predominantly based on qPCR, with the amplification reported by the increasing fluorescent signal from an intercalating dye, dye-labelled primer or labelled probe [1]–[4]. However, qPCR imposes strict requirements on the assay equipment, because of the combined need for temperature cycling, and wavelength-specific fluorescent excitation and emission measurement. These in turn pose limitations through the power consumption and optical arrangements required and have therefore restricted the production of low-cost, simple and robust instruments.

A solution to both the temperature cycling and fluorescence excitation and detection problems is provided by combining alternative amplification methods based on isothermal amplification using strand-displacing polymerases with the bioluminescent reporting of amplification. We show here that a real-time bioluminescent assay, BART can be produced by the simultaneous amplification of a nucleic acid target, conversion of the pyrophosphate produced to ATP, and its determination with a thermostable firefly luciferase. Importantly, this assay can be carried out as a simultaneous combined assay in a single closed tube without further additions, greatly reducing the risk of amplicon contamination of further samples. We further show that such assays can be effectively used on patient-derived samples, that straightforward and cost-effective instruments can be devised for the performance of BART assays, and that they are applicable to RNA targets through coupled reverse transcription.

The BART reporter is unique and clearly distinguishable from any other system used for real-time monitoring of nucleic acid amplification. The characteristic BART bioluminescent signature does not have the sigmoidal shape typical of fluorescent and turbidimetry measurements. It initiates with a high but rapidly declining background signal, followed in the case of a positive sample by a brighter flash and a rapid decline in light intensity. BART curves of this shape were observed not only when amplifying the ChAT template using LAMP, but also using other iNAATs and a range of DNA or viral RNA targets, the latter involving a simultaneous reverse-transcription step with LAMP. The BART light output was found to have the same characteristic shape independent of template, reaction conditions or iNAAT involved, reflecting the exponential production of the amplicon and release of PP_i_. BART assays therefore depend on the coupled amplification technology used, as BART simply reports on any resultant exponential release of PP_i_. We conclude that the dynamics of light output are characteristic of the coupled reactions involved in BART and not any specific amplification.

The high bioluminescent background observed in BART is an inevitable consequence of the reagents required for amplification, but is not problematic for the assay because the BART bioluminescent output reflects the rapid dynamic changes in the relative levels of PP_i_ and ATP. The range of these changes is over two orders of magnitude (0.01–1 mM) and is unique among existing bioluminescent methods. The detectable light output at the beginning of the assay serves as an indicator of BART-reagent viability, and the residual background signal clearly signals non-amplified samples where target nucleic acid is not present. The ability of BART to cope with the presence of dATP, ATP and PP_i_ emphasises its distinction from previous manifestations of ELIDA [7]–[9], [13], which are intolerant to their presence as contaminants and strongly depend on minimising the non-specific background. This tolerance of BART to high background light levels eliminates the need for alternative but less satisfactory solutions such as the use of apyrase to remove ATP, or of d-α-S-ATP, an analogue of dATP which is not a substrate for firefly luciferase and hence does not generate a bioluminescent signal, but which can be incorporated into a nucleic acid, albeit at a much slower rate [33].

Because of the continuous monitoring of light and the measurement of the rate of change of light intensity rather than absolute light levels, BART is quintessentially different from conventional bioluminescent methods based on firefly luciferase in its tolerance to high bioluminescent background, brightness of its light output and quantitation relying on peak timing rather than absolute light intensities. This also provides tolerance to contaminating ATP or PP_i_ from the sample, since rate of change not absolute levels are determined.

Dynamic changes in light intensity are therefore a key feature of BART, allowing analysis to be based on the rate of change of light production rather than absolute light intensity values. A theoretical drawback of BART might be the possible difficulty in distinguishing different sources of PP_i_ production, for example from non-specific processes. However, specific amplification can be differentiated from non-specific by analysing kinetic rates of light output. In non-specific amplification, PP_i_ release is usually slow, non-exponential and not followed by a rapid switch off due to the slower rate. Hence when a wide peak is observed, either with or without a subsequent reduction of the light signal below the background level, it most likely originates from non-specific amplification. We note that the occurrence and frequency of any non-specific amplification is an inherent property of the amplification technology used rather than the BART reporter system, as BART has been found to have no effect on the specificity of amplification. The LAMP-BART combination is particularly favourable as LAMP relies on six primers and eight recognition sites as opposed to the two amplification primers and third detection primer if used in PCR and thereby facilitates a higher specificity of amplification. Nevertheless, assays must be designed and validated to ensure that off-target exponential amplification does not occur, since BART will report on all exponential amplification occurring within the assay, and does not offer the potential for melt-curve analysis or primer binding detection that can be used with qPCR.

We further show that the BART reporter system allows quantitation of the target nucleic acid initially present. It is the only known quantifiable real-time reporter of amplification characterized by time-to-peak rather than by absolute signal output, suggesting a potential greater tolerance of inhibitors or turbid samples resulting from rapid sample preparation methods. The reported profile yields more information than either fluorescence or turbidimetry, both of which generate sigmoid curves. In BART it is possible to derive values for quantitation from either time to the first inflexion point (t_infl_) of the curve or time to its maximal light output (t_max_). Accurate analysis of time to peak can be performed with minimal data processing, and it is easy to compare samples by visual assessment of the raw data. While both parameters can be used for quantitation of the target in a way similar to using Ct values in conventional qPCR, time to the first inflexion may be of particular value in applications where time to result is of the essence.

Detection and measurement of BART signals does not require sophisticated optics or light detection methods. The high tolerance to absolute light intensity means that hardware specifications can tolerate wide variances, enabling low-cost manufacture. The high tolerance to absolute light intensity also widens the range of possible assay volumes. The potential for reducing BART reaction volume without alterations to hardware is practically attractive. BART assays on both instruments described here can be performed in total volumes as low as 2.5 or even 1 µl. The volume reduction results in a lower light output but has no effect on rates of change. Reduction in reaction volume offers savings in reagent costs without sacrificing test parameters and confers potential for miniaturisation. BART cannot be multiplexed conventionally, since if multiple targets of unknown initial concentration are simultaneously amplified, BART will report on the total PP_i_ released from all targets and will be unable to distinguish between them. However the ease and low cost of BART reactions coupled with the flexibility of camera-based equipment to follow large sample numbers through simple image analysis allows multiple reactions to be run simultaneously.

A further advantage that follows from BART's measurements of the kinetics of light output is its robustness to sample contaminants. This includes compounds that could affect luciferase activity, but also the ability of BART to tolerate addition of turbid samples or solid particulates. If the latter causes light absorbance or scattering and reduces absolute light intensity without effecting changes of reaction rates, both qualitative and quantitative analysis of the data are still feasible. This feature is important for molecular diagnostic applications where sample preparation contributes substantially to the cost and time of the whole assay and tolerance to magnetic beads or any other solid particles or pigments represents a significant advantage. Indeed the small trial performed on a panel of human urine specimens demonstrated that LAMP-BART showed robust behaviour, reliably detected *CT* DNA and was not susceptible to inhibition by potential contaminants present in urine-derived samples subjected to rapid DNA preparation.

### Conclusions

BART - the bioluminescent monitoring using coupled conversion of inorganic pyrophosphate to ATP and the simultaneous monitoring of ATP levels using thermostable firefly luciferase - provides an effective system for reporting isothermal nucleic acid amplification in real time. It measures light generated in the process of amplification in a closed tube format and offers the potential for both quantitative and qualitative assays that are simple, fast, robust and low-cost in terms of equipment requirements. BART addresses requirements of molecular diagnostics and is well suited for use in a range of settings and in a wide variety of formats.

## Methods

### Materials and reagents

Unless otherwise noted, chemicals were purchased from Sigma with the exception of luciferin potassium salt (LH_2_; Europa Biotech, Ely, UK), UltraGlow firefly luciferase (UGrLuc; Promega, WI, USA), adenosine-5′-O-phosphosulphate (APS; Biolog Life Science Institute, Bremen, Germany), Bst DNA polymerase large fragment (Bst) and ThermoPol buffer (New England Biolabs, MA, USA), QuantiTech SYBR Green PCR kit (Qiagen, Hilden, Germany), cloned AMV reverse transcriptase and PicoGreen dsDNA Quantitation kit (Invitrogen, CA, USA). Oligonucleotides were synthesized at the Department of Biochemistry, University of Cambridge (UK).

### Template selection and primer design

A 224-base pair (bp) *Chlamydia trachomatis* (*CT*) artificial template (ChAT) was constructed using the Expand High Fidelity PCR System (Roche Applied Science, Indianopolis, IN, USA) from two overlapping oligonucleotides with a 25 bp overlap that reproduces a unique sequence from *CT* cryptic plasmid ORF8 (Genbank accession NC_001372; positions 1088–1311) which is identical in all *CT* strains harbouring this gene. A 224-base pair fragment was cloned into pCR2.1 Topo vector (Invitrogen). Sequences of six oligonucleotides including two Lamp, two loop and two displacing primers designed against the same sequence as described in [19] are shown in Table 3.

**Table 3:** Sequences of six ChAT primers.

Name	Sequence
LampB	gaccgaaggtactaaacaagtttttttgtttaggaatcttgttaagg
LampF	cgcatctaggattagattagattttattggtctattgtccttgg
LoopB	cgagcagcaagctatatt
LoopF	aaactcttgcagattcata
DisplB	tattccttgagtcatcc
DisplF	gatcatatcgaggatctt

### LAMP-BART assay

Optimised LAMP-BART reagent contained 0.2 µM of each displacing primer, 0.4 µM each loop primer, 0.8 µM each LAMP primer, 200 µM each dNTP, 0.16 U/µl Bst DNA polymerase large fragment, 100 µg/ml LH_2_, 100 uM APS, 0.5 U/ml ATP sulfurylase, 5.6–6.2 µg/ml UGrLuc, 60 mM KCl, 0.4 mg/ml polyvinylpyrrolidone and 10 mM DTT in 1× ThermoPol buffer (final concentrations in assay tube are given). BART reactions were run at 55°C in 20 µl total volume containing 15 µl reagent mix with 5 µl added template solution unless otherwise stated. Template was pre-denatured (5 min, 95°C). Reaction mixtures were covered with mineral oil to prevent evaporation. Each sample was run in triplicate.

### Hardware

The LAMP-BART assay was carried out on an assembled instrument comprising a PC-controlled TRobot thermocycler (Biometra, Göttingen, Germany) placed beneath a CCD camera within a ‘Chemi Genius Bio Imaging System’ (Syngene, Cambridge, UK; Figure 6A). This allowed the light emissions to be quantified at any position on the 96-well heating block and measure simultaneously from either a 0.2 ml PCR tubes, 8-well strips or a 96-well PCR plate. Light was integrated over a 60 second intervals using custom software ‘ReactIVD’ (Synoptics, Cambridge, UK) and data saved as images, graphs and Excel spreadsheets.

### LAMP-BART kinetics

1 pg and 100 pg of the ChAT plasmid were run in 50 µl LAMP-BART reactions at 55°C. Full BART kinetic curves from each ChAT concentration were recorded over 60 min. One sample of each ChAT concentration was taken out and placed on ice at 0, 10, 20, 25, 30, 35, 40, 45, 50, 55 and 60 min. DNA concentration in collected samples was determined using PicoGreen® dsDNA Quantitation Kit (Qiagen, Hilden, Germany) following the manufacturer's protocol using a Cary Eclipse fluorimeter (Varian, CA, USA), or by gel electrophoresis run on 2% agarose gels with ethidium bromide visualisation.

### LAMP-BART detection of *Chalmydia trachomatis* in clinical specimens

Analytical specificity and microbial range of LAMP-BART were tested on DNA from 14 different strains of *CT* (Table S1) and from a panel of 28 other pathogenic bacteria and commensals from the oropharynx and genital tract (Table S2). LAMP-BART reactions were run with 2–20 pg DNA. Bacterial DNA was isolated from 0.5 ml urine specimens obtained upon approval of the NHS Research Ethics Committee from 105 patients presenting to the Genitourinary clinic at Addenbrooke's Hospital (Cambridge, UK) using ChargeSwitch gDNA Mini Bacteria Kit (Invitrogen, CA, USA) following the manufacturer's protocol. The data were analyzed anonymously and did not require patient consent. All urine specimens were either stored at +4°C for ≤5 days or frozen within 5 days of collection and stored at −20°C. Each sample was analysed in duplicate by BART and in-house qPCR in parallel with a ChAT dilution series used for calibration. qPCR was run in 10 µl reactions containing 1× QuantiTech SYBR Green PCR reagent, 0.4 µM forward and reverse primers (TTCCTTGAGTCATCCTGTTTAGG and TTGTCCTTGGATATGAATCTGC, respectively) and 2.5 µl of the sample. qPCR was run on Rotor Gene 3000 (Corbett Research, Australia) using the following profile: 10 min at 94°C, then 50 cycles of 30 s at 94°C, 30 s at 56°C, 45 s at 72°C, then 15 s at 72°C and a melt step.

### LAMP-BART comparison with TaqMan PCR

*CT* QCMD 2010 panel (Qnostics, UK) was used in the side-by-side comparison of ChAT LAMP-BART and TaqMan PCR. Samples were resuspended in 200 µl of molecular grade water (except for sample 5 resuspended in 1 ml). 200 µl of each sample were extracted using Qiagen DX Reagent Pack on Corbett Robotics Extractor connected to a vacuum pump. Extracted DNA was eluted in 100 µl of molecular grade water. Fully evaluated TaqMan PCR used for *CT* routine screening of urine and swab clinical specimens at Health Protection Agency laboratories in Cambridge, UK, was carried out by introducing 12 µl of extracted samples to 14 µl of the reagent [31]. qPCR was run on Rotor Gene 6000 (Qiagen, Germany). To make target loads identical between the amplification assays the volumes of sample and reagent used in LAMP-BART were adjusted to 12 and 14 µl, respectively, with the final concentrations of all ingredients remaining as described above. LAMP-BART was run at 60°C for 90 min.

### RT-LAMP-BART of purified *classic swine fever RNA*

A pGEM construct containing 163-base pair (bp) DNA fragment complementary to the classic swine fever viral RNA (CSFV) sequence was a kind gift from Friedrich-Loeffler-Institut (Germany). Sequences of the CSFV target and five oligonucleotides including two Lamp, two loop and one displacing primer designed against the sequence as described in [19] are shown in Table 4.

**Table 4:** Sequences of the CSFV target and five CSFV primers.

Name	Sequence
Target	atgcccacagtaggactagcaaacggagggactagccgtagtggcgagctccctgggtggtctaagtcctgagtacaggacagtcgtcagtagttcgacgtgagcagaagcccacctcgatatgctatgtggacgagggcatgcccaagacacaccttaaccc
LampB	ccacccagggagctcgccacttttatgcccacagtaggactagc
LampF	cgtcagtagttcgacgtttttggcatgccctcgtccacatagc
LoopB	ctagtccctccgtttgc
LoopF	gagcagaagcccacctcg
DisplB	gggttaaggtgtgtcttg

Displacing primer B was omitted because the target sequence was too short to accommodate a full set of six primers.

RNA was in-vitro transcribed from the pGEM construct using AmpliScribe™ T7 High Yield Transcription Kit (EPICENTRE Biotechnologies, Madison, USA) according to the manufacturer's recommendations. To achieve full removal of DNA the mixture was treated twice with RNAse free DNAse. Quality and concentration of RNA preparations was assessed spectrophotometrically using a Nanodrop spectrophotometer (Thermo Scientific).

RT-LAMP-BART reagent contained 0.4 µM of displacing primer F, 0.8 µM each loop primer, 1.6 µM each LAMP primer, 300 µM each dNTP, 0.16 U/µl Bst DNA polymerase large fragment, 1.5 U/µl cloned AMV reverse transcriptase, 100 µg/ml LH_2_, 100 uM APS, 0.5 U/ml ATP sulfurylase, 5.6–6.2 µg/ml UGrLuc, 50 mM KCl, 0.4 mg/ml polyvinylpyrrolidone and 10 mM DTT in 1× ThermoPol buffer (final concentrations in assay tube are given). BART reactions were run at 55°C in 20 µl total volume containing 15 µl reagent mix with 5 µl added template solution. Reaction mixtures were covered with mineral oil to prevent evaporation. Each sample was run in triplicate.

## Supporting Information

Table S1 Chlamydia strains tested for inclusivity.
(0.03 MB DOC)

Table S2 Pathogenic bacteria and commensal organisms of the oropharynx and genital tract tested for cross-reactivity.
(0.04 MB DOC)

Movie S1 LAMP-BART of a ChAT dilution series: red - 1 ng, orange - 100 pg, green - 10 pg, blue - 1 pg, black - no-template control. Top row - ChAT DNA dilution series with decreasing amount of template (left to right); bottom row - no-template control.
(0.85 MB MOV)
