# Supplementary material for: Novel Bioluminescent Quantitative Detection of Nucleic Acid Amplification in Real-Time
Source: PLoS One. 2010 Nov 30;5(11):e14155. doi: 10.1371/journal.pone.0014155 (PMC2994769; doi:10.1371/journal.pone.0014155)
Supplement: Table S1 — Chlamydia strains tested for inclusivity. (0.03 MB DOC) [file pone.0014155.s001.doc]

| **Strain** | **ATCC Code** |
| --- | --- |
| A strain HAR-13 | VR-571B |
| B strain HAR-36 | VR-573 |
| C strain TW-3 | VR-1477 |
| D strain UW-36/Cx | VR-885 |
| E strain BOUR | VR-348B |
| F strain IC-Cal-3 | VR-346 |
| G strain UW-57/Cx | VR-878 |
| H strain UW-43/Cx | VR-879 |
| I strain UW-12/Ur | VR-880 |
| J strain UW-36/Cx | VR-886 |
| K strain UW-31/Cx | VR-887 |
| LGV I strain 440 | VR-901B |
| LGV II strain 434 | VR-902B |
| LGV III strain 404 | VR-903 |
