# Supplementary material for: Novel Bioluminescent Quantitative Detection of Nucleic Acid Amplification in Real-Time
Source: PLoS One. 2010 Nov 30;5(11):e14155. doi: 10.1371/journal.pone.0014155 (PMC2994769; doi:10.1371/journal.pone.0014155)
Supplement: Table S2 — Pathogenic bacteria and commensal organisms of the oropharynx and genital tract tested for cross-reactivity. (0.04 MB DOC) [file pone.0014155.s002.doc]

| *Streptococcus mutans* | ATCC 25175 |
| --- | --- |
| *Streptococcus canis* | ATCC 43497 |
| *Streptococcus sanguis* | ATCC 10556 |
| *Staphylococcus aureus* | ATCC 25923 |
| *Staphylococcus epidermidis* | ATCC 12228 |
| *Neisseria meningitidis* | ATCC 13077 |
| *Neisseria gonorrhoeae* | NCTC 8375 |
| *Escherichia coli* | NCTC 10418 |
| *Cornyebacterium diphtheriae* | ATCC 11913 |
| *Cornyebacterium ulcerans* | NCTC 7907 |
| *Pasteurella haemolytica* | NCTC 9380 |
| *Yersinia enterocolitica* | ATCC 23715 |
| *Arcanobacterium haemolyticum* | NCTC 8452 |
| *Prevotella melaninogenica* | ATCC 25845 |
| *Actinomyces israelii* | ATCC 10049 |
| *Clostridium difficile* | NCTC 11209 |
| *Acholeplasma laidlawii* | NCTC 10116 |
| *Mycoplasma fermentans* | NCTC 10117 |
| *Mycoplasma hyorhinis* | NCTC 10130 |
| *Mycoplasma orale* | NCTC 10112 |
| *Mycoplasma hominis* | NCTC 10111 |
| *Prevotura buccae* | ATCC 33574 |
| *Veillonella parvula* | ATCC 10790 |
| *Fusobacterium necrophorum* | NCTC 10576 |
| *Fusobacterium ulcerans* | NCTC 12111 |
| *Fusobacterium nucleatum* | ATCC 25586 |
| *Fusobacterium necro ssp. fundiliforme* | ATCC 51357 |
| *Haemophilis actinomycetemcomitans* | ATCC 29522 |
